# Supplementary material for: Reversible bipolar thermopower of ionic thermoelectric polymer composite for cyclic energy generation
Source: Nat Commun. 2023 Jan 19;14:306. doi: 10.1038/s41467-023-36018-w (PMC9852232; doi:10.1038/s41467-023-36018-w)
Supplement: Supplementary file 1 — Supplementary Information [file 41467_2023_36018_MOESM1_ESM.pdf]

Supplementary Information

Reversible Bipolar Thermopower of Ionic Thermoelectric Polymer Composite for Cyclic Energy Generation

Cheng Chi[1,2†], Gongze Liu[3†], Meng An[1,4†], Yufeng Zhang[1], Dongxing Song[1], Xin Qi[1], Chunyu Zhao[1], Zequn Wang[4], Yanzheng Du[1], Zizhen Lin[1], Yang Lu[1], He Huang[3], Yang Li[3], Chongjia Lin[3], Weigang Ma[1*], Baoling Huang[3*], Xiaoze Du[2], and Xing Zhang[1]

[1]Key Laboratory for Thermal Science and Power Engineering of Ministry of Education, Department of Engineering Mechanics, Tsinghua University, Beijing, 100084, China.

[2]Key Laboratory of Power Station Energy Transfer Conversion and System of Ministry of Education, School of Energy Power and Mechanical Engineering, North China Electric Power University, Beijing 102206, China.

[3]Department of Mechanical and Aerospace Engineering, The Hong Kong University of Science and Technology, Clear Water Bay, Hong Kong SAR, China.

[4]College of Mechanical and Electrical Engineering, Shaanxi University of Science and Technology, Xi'an, 710021, China.

*Corresponding author email: maweigang@tsinghua.edu.cn (W. G. Ma); mebhuang@ust.hk (B. L. Huang).

[†]The authors contribute equally.

Inventory of Supplementary Information:

Supplementary Figures

Supplementary Fig. S1 The illustration of the measurement setup of ionic thermopower.

Supplementary Fig. S2 The measured thermal voltages using Cu and a-CNT electrodes.

Supplementary Fig. S3 The XPS study of the surface property of Cu electrode.

Supplementary Fig. S4 The control experiment by noble electrodes.

Supplementary Fig. S5 The morphology of various CNTs.

Supplementary Fig. S6 The humidity effect on the *p*-type Cu|PNP|Cu.

Supplementary Fig. S7 The humidity effect on the *n*-type a-CNT|PNP|a-CNT.

Supplementary Fig. S8 The Raman spectra of the materials.

Supplementary Fig. S9 The study of interfacial effect by the Raman measurement.

Supplementary Fig. S10 The surface morphology comparison of Cu and a-CNT electrodes.

Supplementary Fig. S11. The illustration of the simulation models.

Supplementary Fig. S12 The density number of the ions near the electrodes.

Supplementary Fig. S13 The study of the interfacial interaction between ions and electrodes.

Supplementary Fig. S14 The thermopower test setup of the a-CNT|PNP|Cu system.

Supplementary Fig. S15 The temperature effect on the a-CNT|PNP|Cu system.

Supplementary Fig. S16 The ion distribution of the a-CNT|PNP|Cu system.

Supplementary Fig. S17 The thermopower of the a-CNT|PNP|Cu system.

Supplementary Fig. S18 The demo of the ionic thermoelectric generator.

Supplementary Fig. S19 The fabrication process flow of the ionic thermoelectric sensor.

Supplementary Fig. S20 The working principle of the ionic thermoelectric sensor.

Supplementary Table S1 The comparison of the physical property of various CNTs

Supplementary Methods

Supplementary References

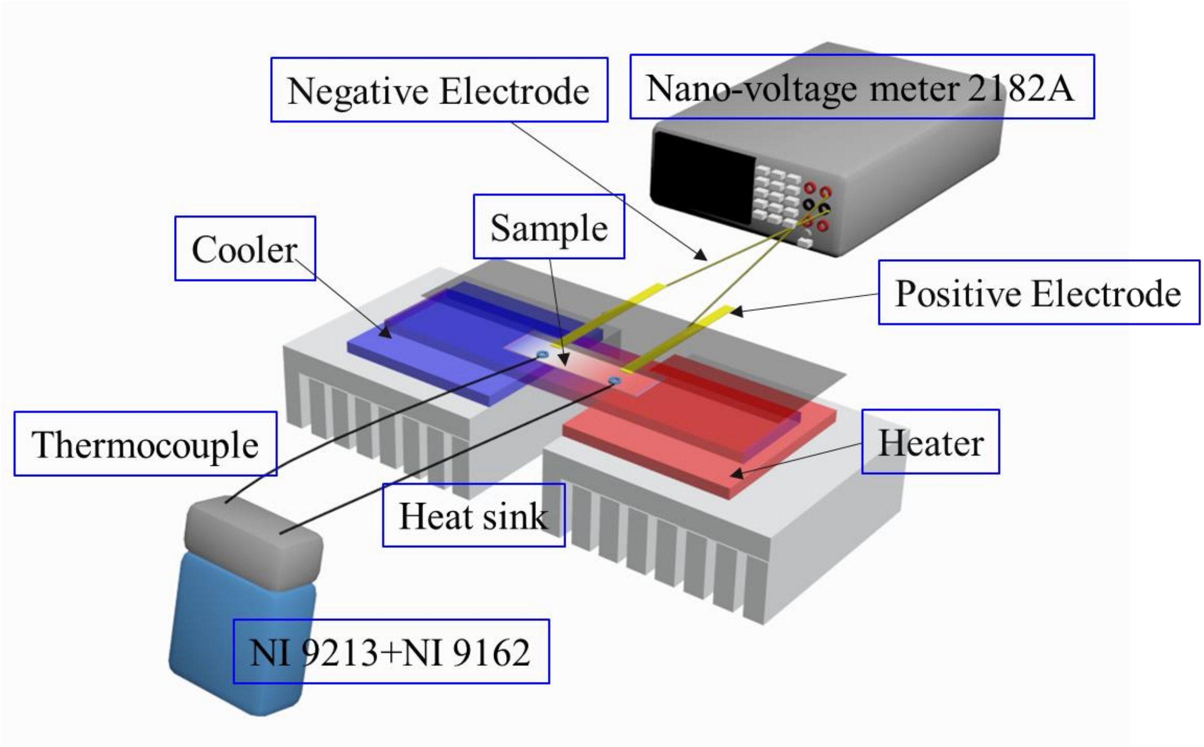

Fig. S1 The illustration of the measurement setup of ionic thermopower.

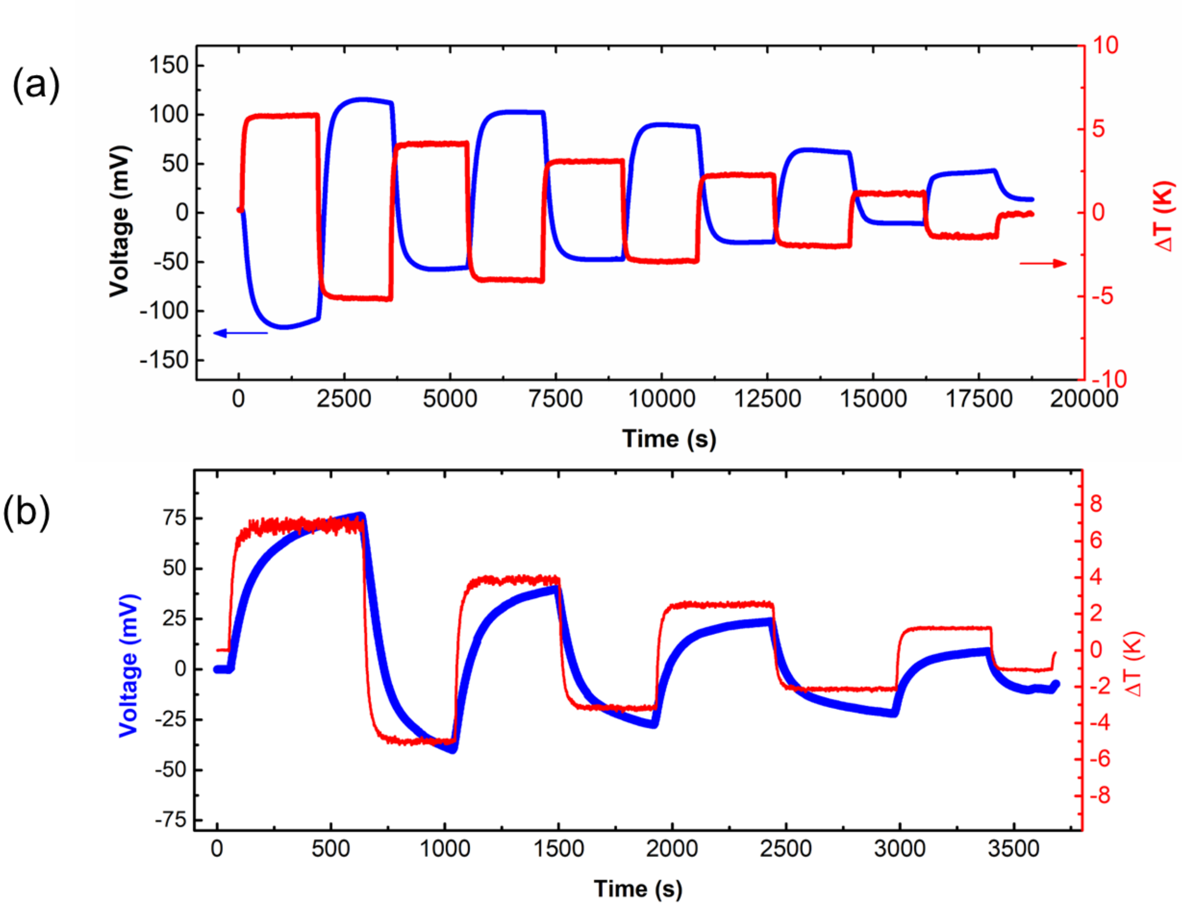

Fig. S2 The measured thermal voltages using Cu and a-CNT electrodes. The measured thermal voltage of (a) Cu|PNP|Cu and (b) a-CNT|PNP|a-CNT under positive and negative temperature difference ΔT (red line)

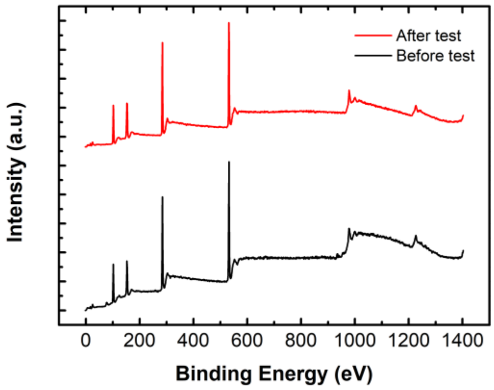

Fig. S3 The XPS study of the surface property of Cu electrode. The XPS characterization of the surface of the Cu electrode before and after thermopower measurement.

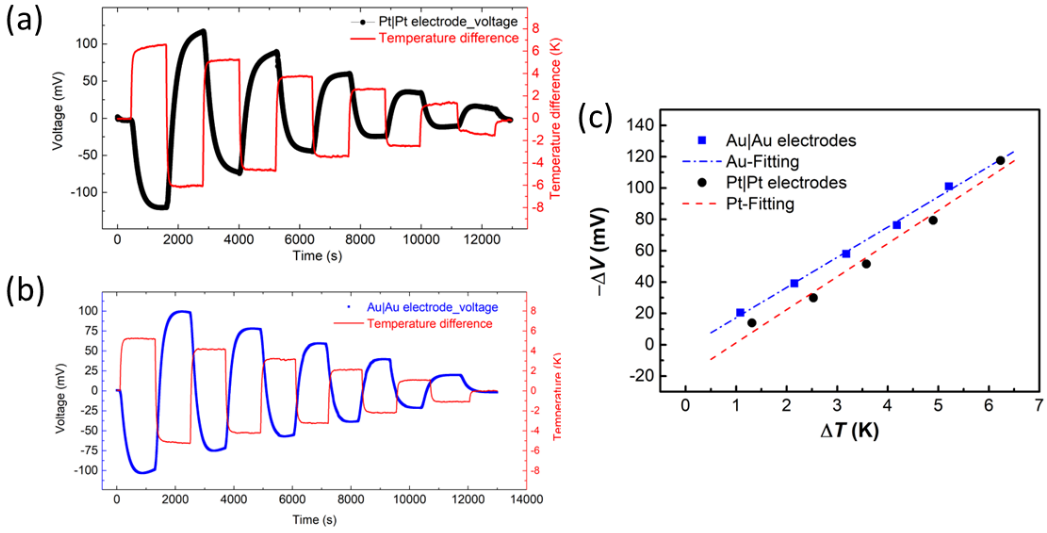

Fig. S4 The control experiment by noble electrodes. The measured thermal voltage curves of (a) Pt|PNP|Pt system and (b) Au|PNP|Au system as a function of time. (c) The fitting curves -(V_{hot}-V_{cold})/ΔT of the Pt|PNP|Pt, and Au|PNP|Au systems.

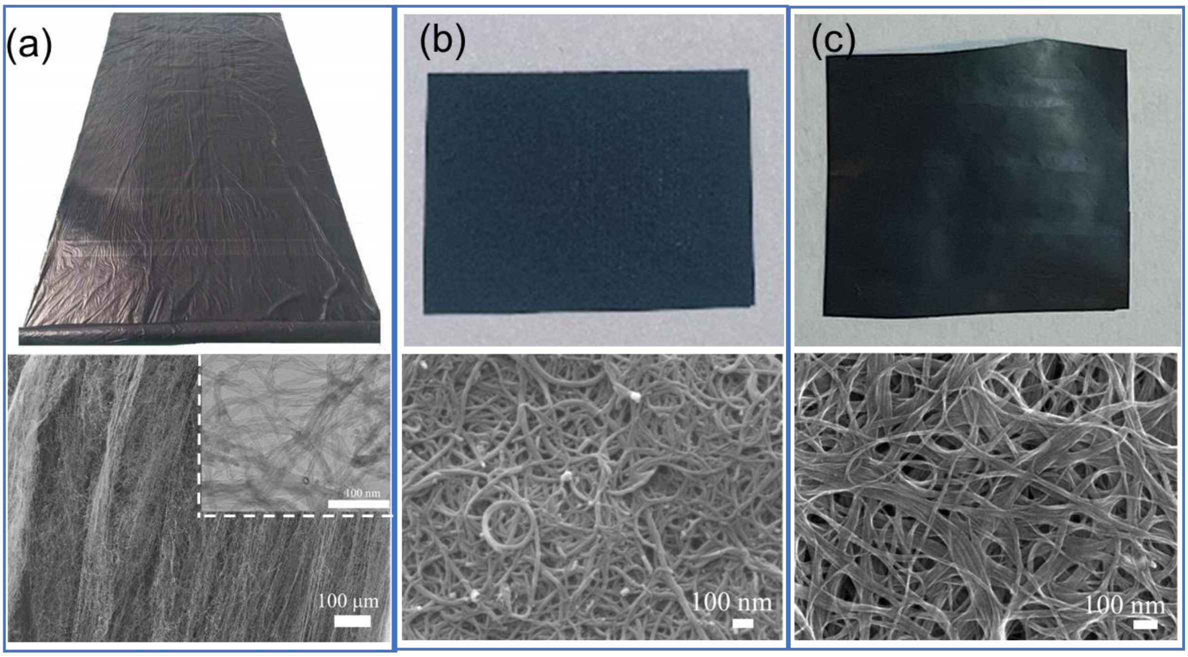

Fig. S5. The morphology of various CNTs. The digital and SEM images of (a) aligned CNTs, (b) MWCNTs, and (c) SWCNTs electrodes.

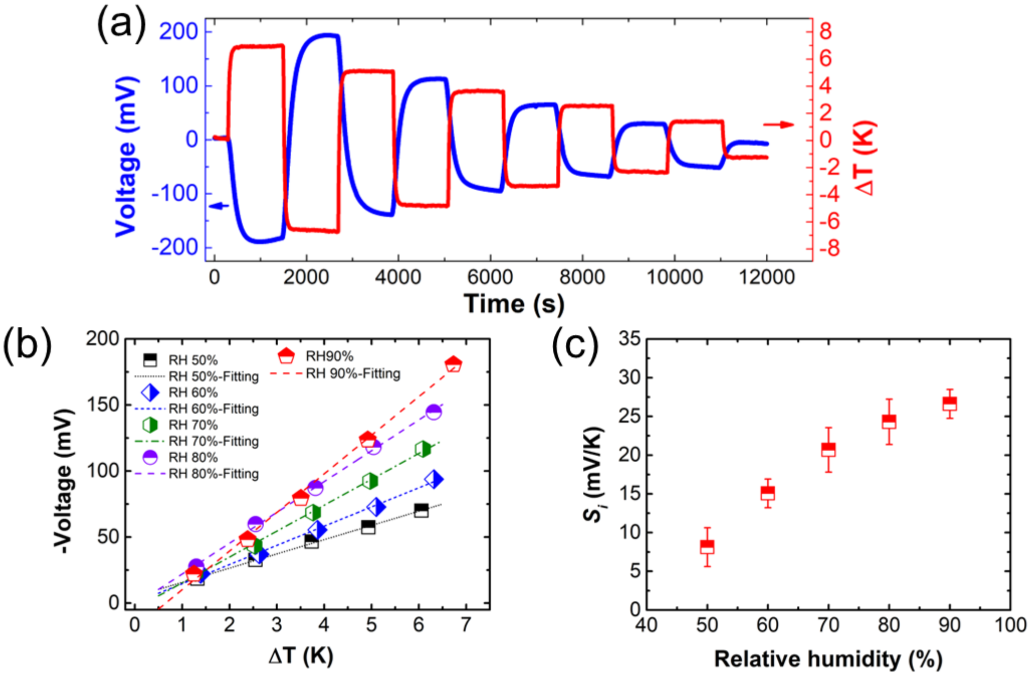

Fig. S6 The humidity effect on the *p*-type Cu|PNP|Cu. (a) The measured thermoelectric voltage at a series of temperature differences of Cu|PNP|Cu sample at RH 90%, (b) the plot of –(Δ*V*)-Δ*T* fitting curves, and (c) the thermopower of Cu|PNP|Cu sample various humidity ranging from RH 50% to RH 90%.

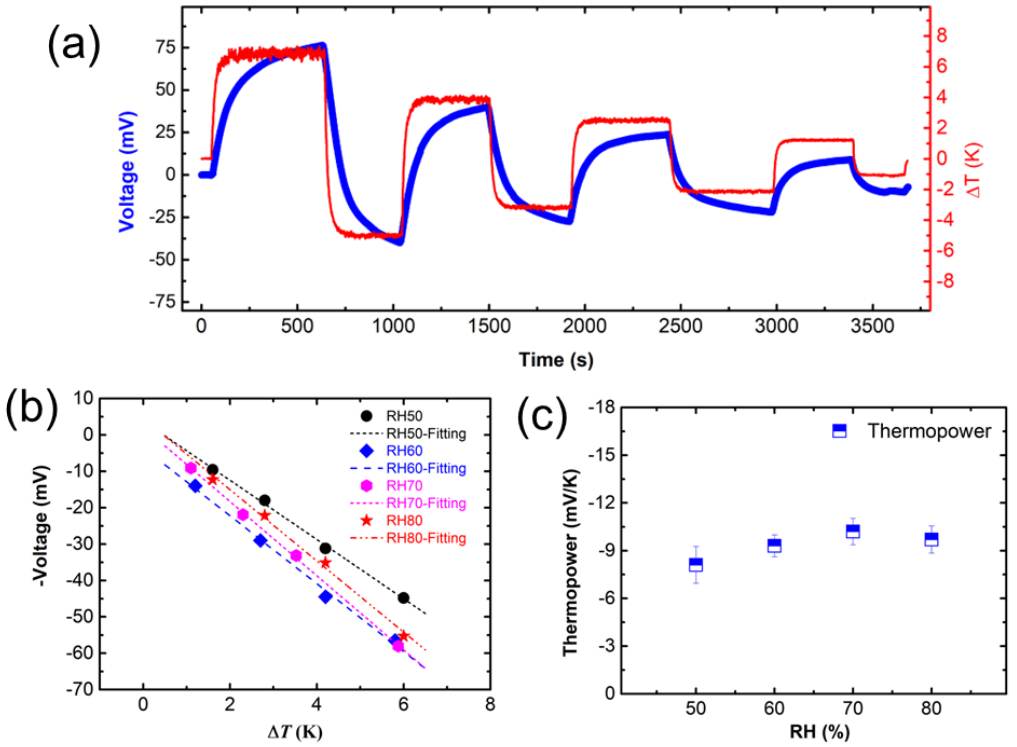

Fig. S7. The humidity effect on the *n*-type a-CNT|PNP|a-CNT. (a) The measured thermoelectric voltage at a series of temperature differences of a-CNT|PNP| a-CNT sample at RH 70%, (b) the plot of $-(\Delta V)$-ΔT fitting curves, and (c) the thermopower of a-CNT|PNP|a-CNT sample various humidity ranging from RH 50% to RH 80%.

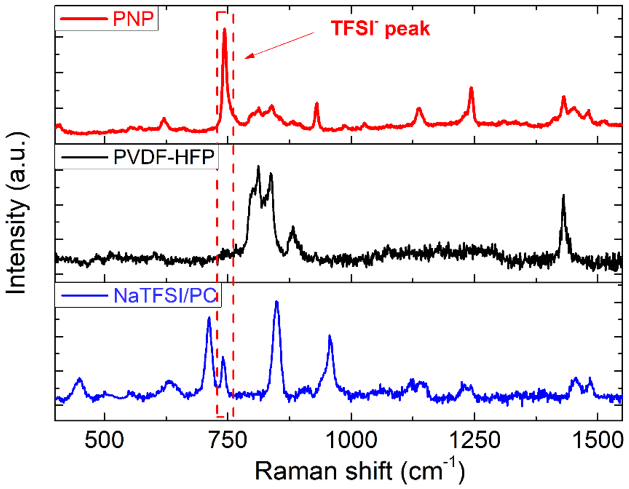

Fig. S8 The Raman spectra of the materials. (a) PNP, (b) PVDF-HFP, and (c) NaTFSI/PC.

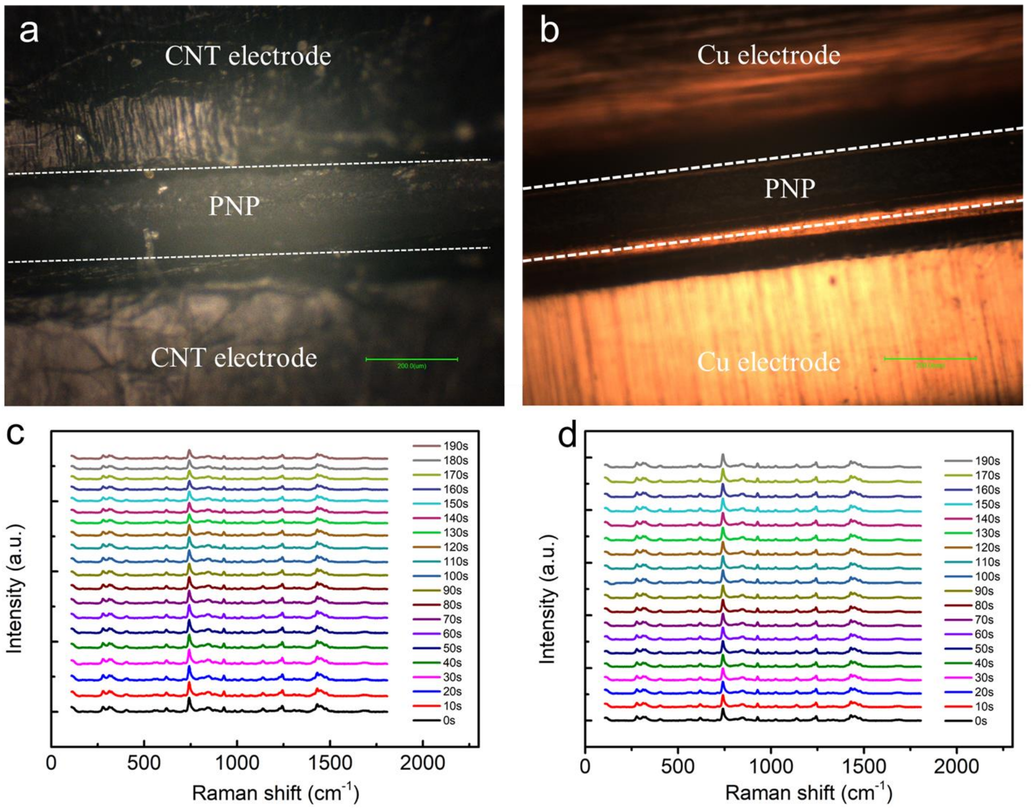

Fig. S9 The study of interfacial effect by the Raman measurement. The images of the cross-section view of the (a) a-CNT|PNP|a-CNT and (b) Cu|PNP|Cu system. Line-scanning Raman spectra of near (c) a-CNT and PNP interface of a-CNT|PNP|a-CNT system and (d) the Cu-PNP interface of the a-CNT|PNP| a-CNT system in a range of 100-1800 cm^{-1}.

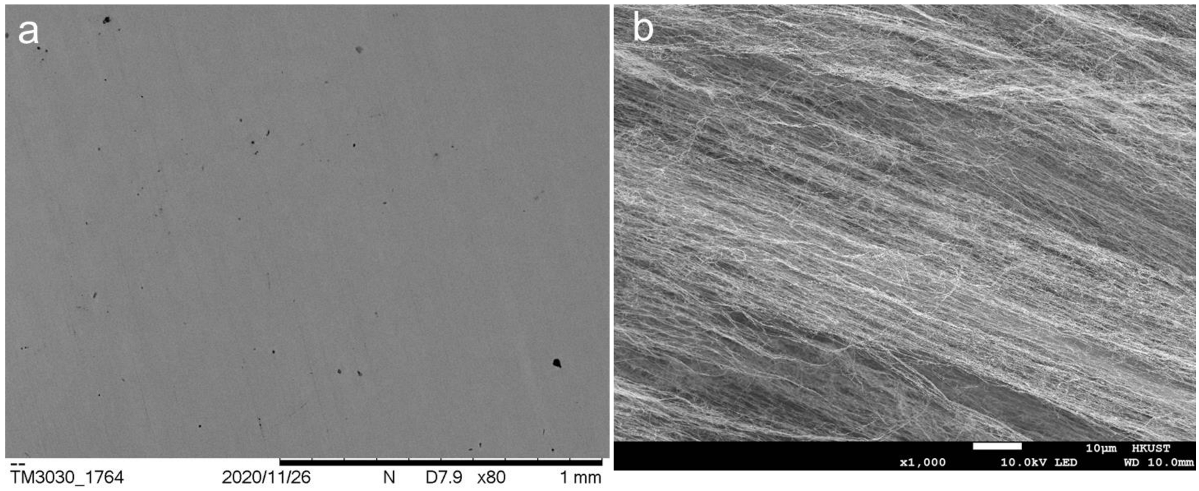

Fig. S10 The surface morphology comparison of Cu and a-CNT electrodes. The SEM of the surface morphology of the (a) Cu and (b) a-CNT electrodes.

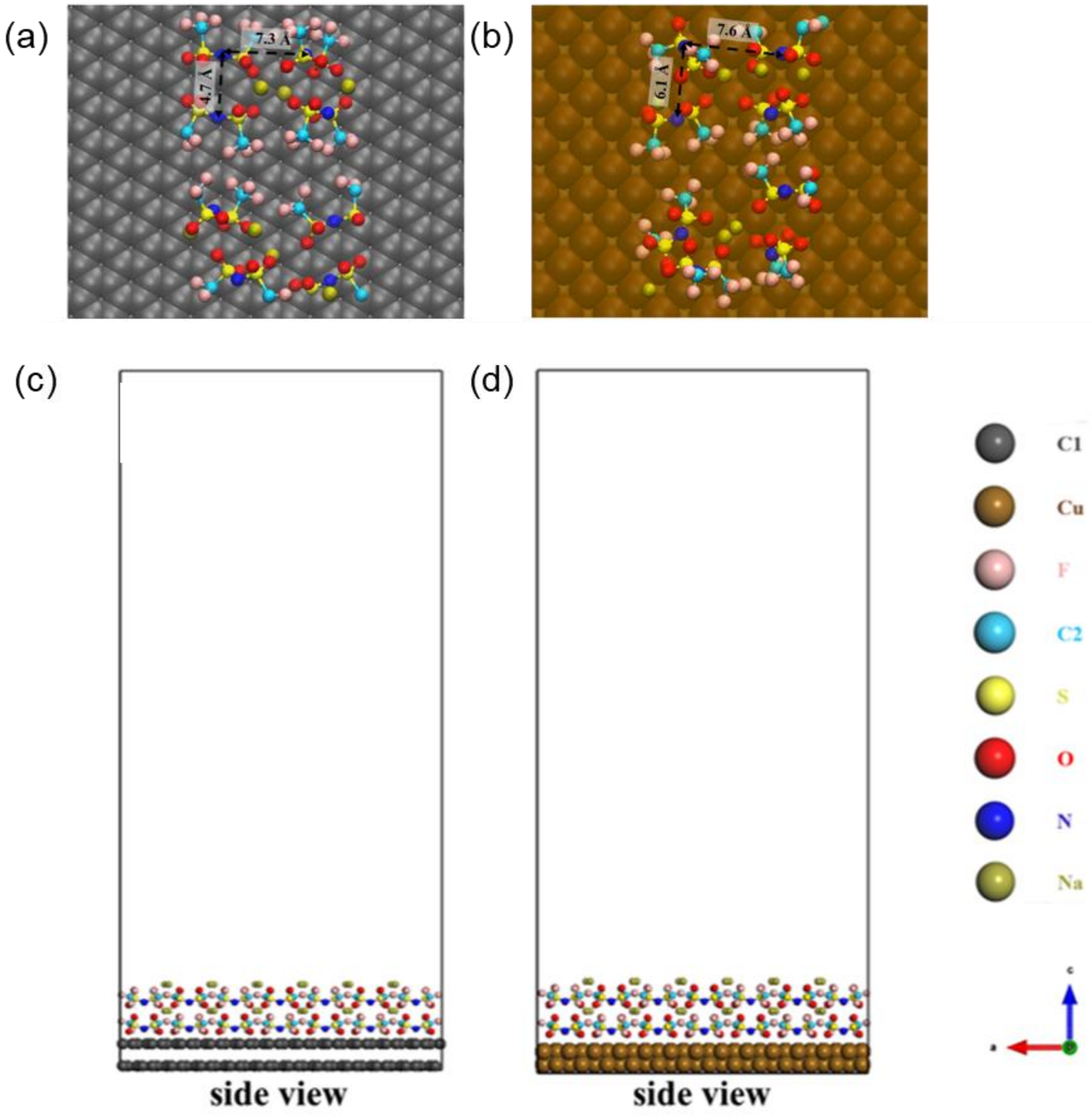

Fig. S11 The illustration of the simulation models. The top view of the NaTFSI on (a) a-CNT electrode surfaces and (b) Cu surfaces. The side view of the NaTFSI on (c) a-CNT electrode surfaces and (d) Cu surfaces.

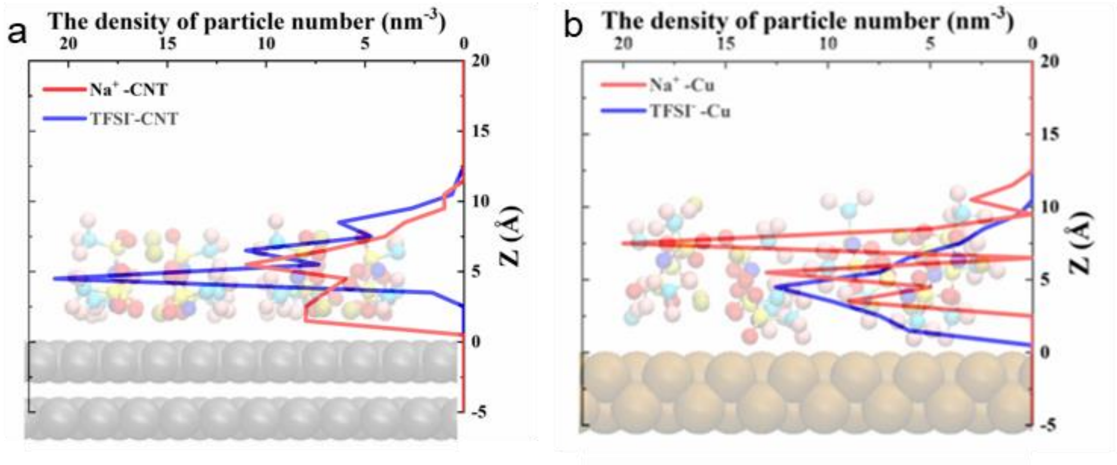

Fig. S12 The density number of the ions near the electrodes. The side view of the MD snapshot of the density profile of Na^+ and $TFSI^-$ ions along the *Z* direction (a) on the a-CNT electrodes and (b) on the Cu electrodes.

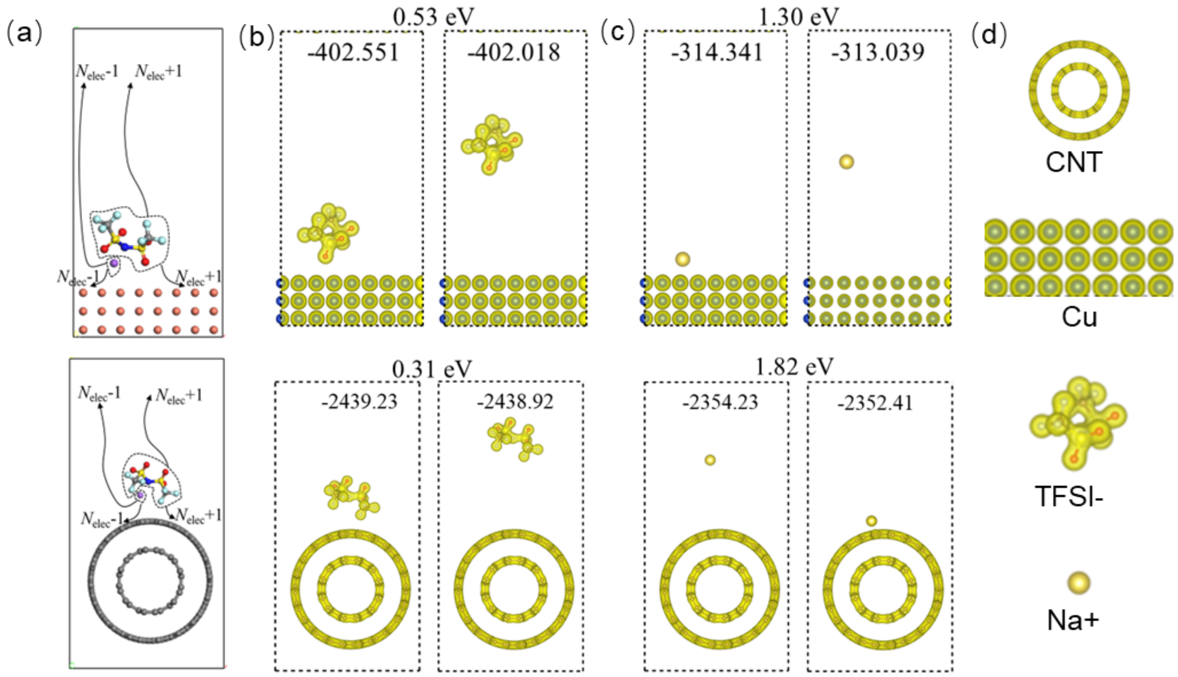

Fig.S13 The study of the interfacial interaction between ions and electrodes. (a) The interface models between electrodes and ions in both the Cu|PNP|Cu, and a-CNT|PNP|a-CNT systems. The interfacial interaction energy between (b) Cu-TFSI$^-$ ions (top) and a-CNT-TFSI$^-$ ions (bottom), and (c) Cu-Na$^+$ ions (top) and a-CNT-Na$^+$ ions (bottom). (d) The illustration of the symbols of the electrodes and ions.

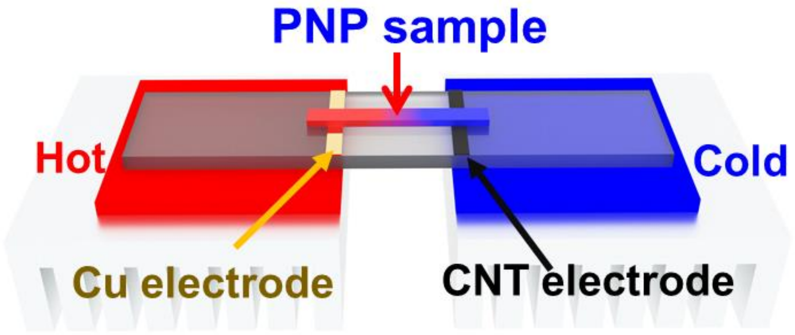

Fig. S14 The thermopower test setup of the a-CNT|PNP|Cu system.

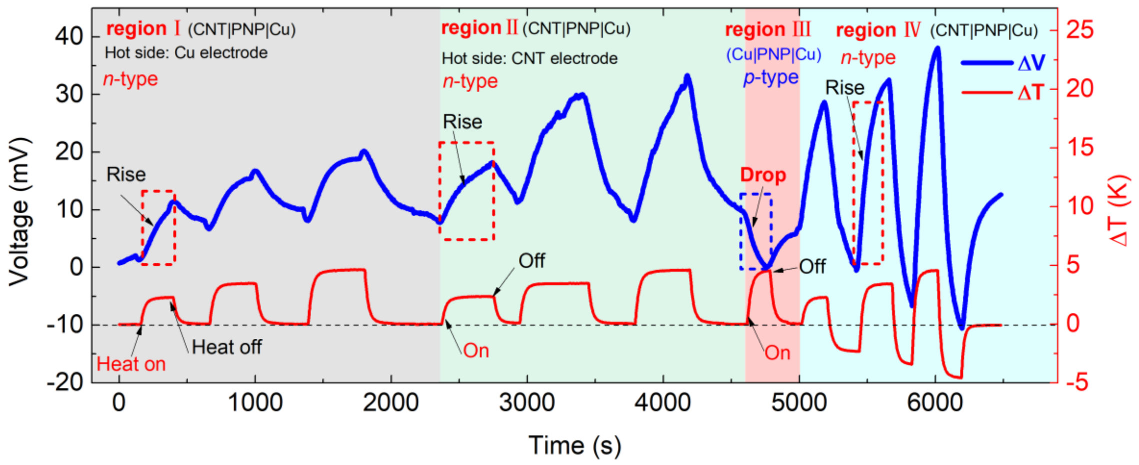

Fig. S15 The temperature effect on the a-CNT|PNP|Cu system. The measured voltage as a function of the time of the a-CNT|PNP|Cu system under various test temperature differences.

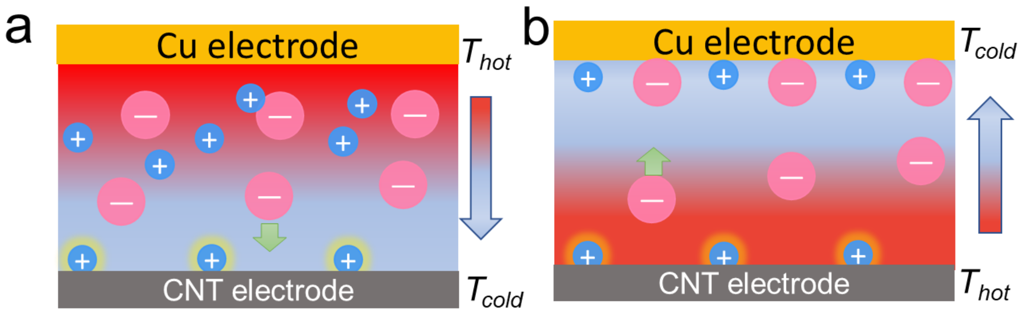

Fig. S16 The ion distribution of the a-CNT|PNP|Cu system. (a) Heating the Cu electrode side and (b) heating the a-CNT electrode side.

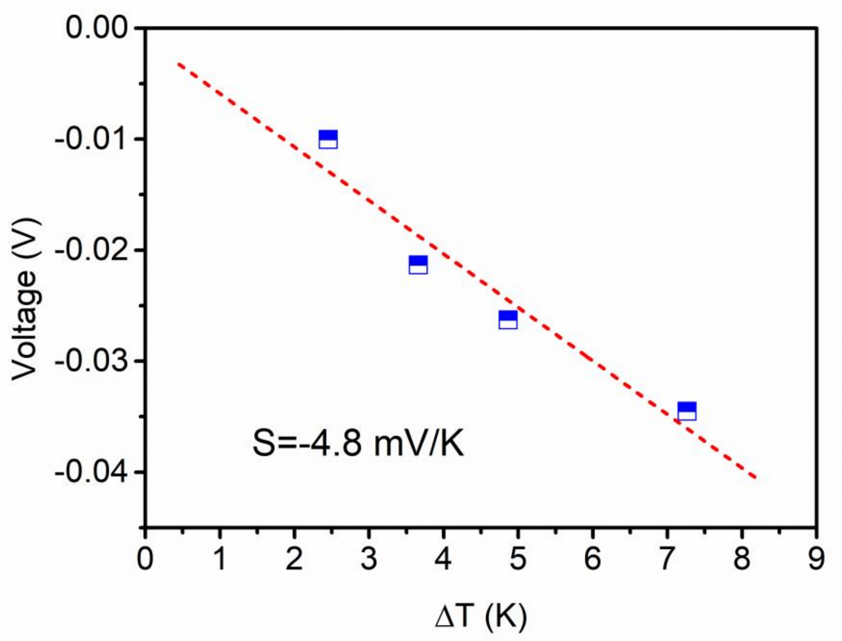

Fig. S17 The thermopower of the a-CNT|PNP|Cu system. The Δ*V vs t* curve of the a-CNT|PNP|Cu system.

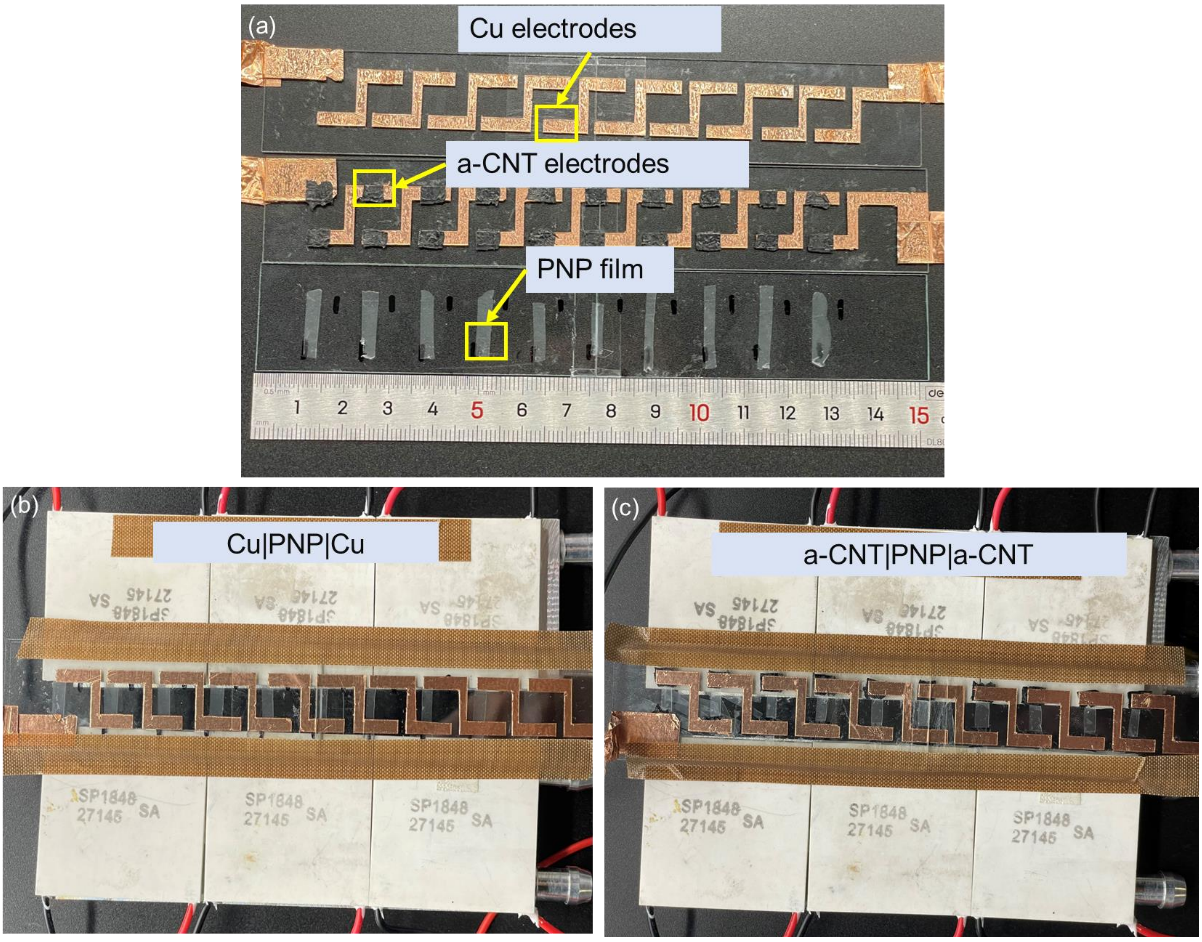

Fig. R18. The demo of the ionic thermoelectric generator. (a) The digital photo of the fabricated *i*-TEG by 10 pieces of PNP films alternatively contacting with (b) Cu|Cu electrodes and (c) a-CNT|a-CNT electrodes.

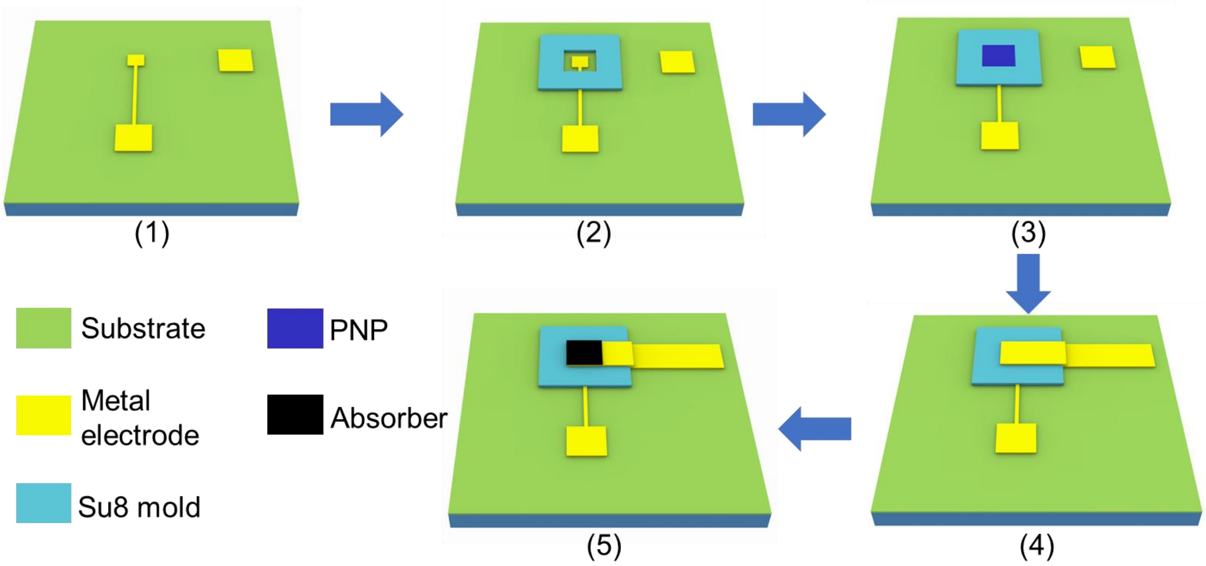

Fig. S19 The fabrication process flow of the ionic thermoelectric sensor.

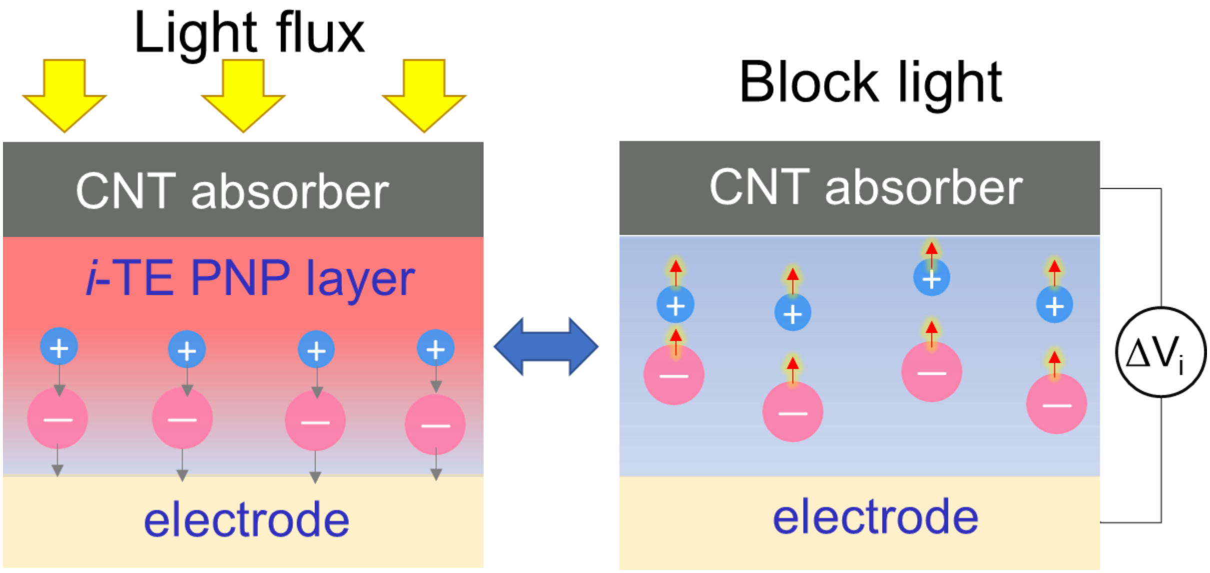

Fig. S20 The working principle of the ionic thermoelectric sensor.

Table S1 The comparison of the physical property of various CNTs.

Property	Thickness	Conductivity	Sheet resistance	Strength	Length	Diameter
Aligned CNT	~10 μm	~3×10^5 S m^{-1}	1~1.5 Ω m^{-2}	~120 MPa	continuous growth	20-50 nm
MWCNT	~0.15 mm ± 0.05mm	/	~4 Ω m^{-2}	~10 MPa	10-30 μm	20-30 nm
SWCNT	60-80 μm	2~4×10^3 S m^{-1}	/	10-15 MPa	10-30 μm	1-2 nm

Supplementary Methods

Preparation of PNP film

The PVDF-HFP pellets were firstly dried in an oven at 100 ℃ for 24 h and were dissolved in NMP (N-Methylpyrrolidone) at a concentration of 0.1 g mL^{-1} with rapid magnetic stirring for about 8 h at 60 ℃. Next, the NaTFSI and PC were added to the PVDF-HFP to form a homogeneous mixture in an argon atmosphere. The resulting mixture was then cast on a glass petri-dish and dried in a vacuum oven (10^{-3} Torr) at 60 ℃ for over 1~10 h to obtain the free-standing PNP films. All the films were stored in a glove box (O_2 < 0.1 ppm, and H_2O < 0.1 ppm) for future use.

Electrodes

The a-CNT, MWCNTs, and SWCNT films were purchased from Nanjing Ji Cang Nano Technology Co., Ltd. (Nanjing, China). The a-CNT film was fabricated by the chemical vapor deposition (CVD) method and continuously grown into a large-scale thin film, of which the product number is JCNTF-20C.

Thermopower measurement

The tested *i*-TE materials with a rectangular shape were suspended on two electrodes with a separation distance of several tens of millimeters (Fig. S1). And one Peltier heater and one Peltier cooler were located under the tested materials, powered by two Keithley 2400 source meters, providing temperature difference along the length direction of PNP. The produced thermal voltages by the *i*-TE materials were recorded with a nano voltage meter (Keithley 2182A). Two T-type thermocouples were applied to record temperature variation between the hot and cold sides of *i*-TE materials controlled by NI 9213 coupled with NI 9162 modes. The whole test system was first calibrated well with the reported *i*-TE materials in the previous work.

The fabrication process of the i-TE sensor.

Step 1: 20 nm Ti with 150nm Al thick metal bottom electrode was formed on a substrate utilizing a lift-off process.

Step 2: The 10 μm thick SU8 mold was aligned with the bottom electrode and fabricated by photolithography.

Step 3: The PNP was filled into SU8 mold by drop-casting or spin coating.

Step 4: The 20 nm Ti layer with a 150nm Al thick metal top electrode is formed by a sputtering process with a shadow mask aligned with PNP.

Step 5: The absorber layer is fabricated by the spin coating or spray coating on top of the device with a shadow mask.

Characterization

A scanning electron microscope (SEM, JEOL-7100F) and Transmission Electron Microscope (TEM, JEOL 2010F) were used to characterize the morphology of the materials. In-situ Raman characterization was conducted using (HORIBA LabRAM) within the range of 100-1800 cm^{-1} and a laser source of 633 nm. The X-ray photoelectron spectroscopy (XPS) measurement was conducted using PHI 5600.

All the measurements including thermopower are performed using at least 5 separate samples and the error bars are calculated using standard derivation.

MD details of interfacial adsorption on Cu and a-CNT electrodes

To model the adsorption behavior of NaTFSI ($C_2F_6NNaO_4S_2$) on the electrode surfaces of Cu and a-CNT at the molecular level, the two models are constructed, which are consisted of the interfacial layer of the NaTFSI and electrode surfaces shown in Fig. S4. The basal atoms of a-CNT and Cu electrodes were fixed on a cubic lattice, where the size of the simulation cell is 4.7 nm×4.4 nm ×10.0 nm. The Periodic boundary conditions were employed in two planar directions. All the MD simulations were performed by the large-scale atomic/molecular

massively parallel simulator (LAMMPS) package[1]. The OPLS-all atom (OPLS-AA) force field[2] is used to describe the interaction among atoms in NaTFSI, and the interatomic interactions of a-CNT are described by Tersoff potential[3,4]. The interactions between NaTFSI and basal atoms were described by 12-6 Lennard-Jones potentials[5]. A cutoff distance of 12.0 Å was used for computing long-range interactions of van der Waals (VDW) and the long-range electrostatic forces solved by the particle-particle particle-mesh (PPPM) method, where the convergence parameter of the PPPM method is 10^{-4}. Initially, the simulation systems are relaxed along with two planar directions under the NPT ensemble to relax the internal stress inside the systems. Then the simulations were switched to NVE ensemble for 10 ns and Berendsen thermostat[6] with a time step of 1 fs is applied to keep the system temperature at 300 K. The density and radial distribution function of systems are recorded to analyze the interfacial behavior and its mechanisms for last 9 ns.

Supplementary References

1. Plimpton S. Fast parallel algorithms for short-range molecular-dynamics. *J. Comput. Phys.* **117**, 1-19 (1995).
2. Jorgensen W. D. A. F. J. T. R. W. L. OPLS all-atom force field for carbohydrates. *J. Comput. Chem.*, 1955-1970 (1997).
3. Tersoff J. Modeling solid-state chemistry: Interatomic potentials for multicomponent systems *Phys. Rev. B* **39**, 5566 (1989).
4. Tersoff J. New empirical approach for the structure and energy of covalent systems. *Phys. Rev. B* **37**, 6991 (1988).
5. C. Chi, *et al.* Selectively tuning ionic thermopower in all-solid-state flexible polymer composites for thermal sensing. *Nat. Commun.* **13**, 221 (2022).
6. Haak H. J. C. B. J. P. M. P. W. F. v. G. A. D. J. R. Molecular dynamics with coupling to an external bath. *J. Chem. Phys.* **81**, 3684 (1984).
