# Supplementary material for: Reversible bipolar thermopower of ionic thermoelectric polymer composite for cyclic energy generation
Source: Nat Commun. 2023 Jan 19;14:306. doi: 10.1038/s41467-023-36018-w (PMC9852232; doi:10.1038/s41467-023-36018-w)
Supplement: Supplementary file 3 — Description of Additional Supplementary Files [file 41467_2023_36018_MOESM3_ESM.pdf]

Description of Additional Supplementary Files

Supplementary Movie 1: The demonstration of the i-TE sensor
